# Re-Occupancy of Breeding Territories by Ferruginous Hawks in Wyoming: Relationships to Environmental and Anthropogenic Factors

**DOI:** 10.1371/journal.pone.0152977

**Published:** 2016-04-06

**Authors:** Zachary P. Wallace, Patricia L. Kennedy, John R. Squires, Robert J. Oakleaf, Lucretia E. Olson, Katie M. Dugger

**Affiliations:** 1 Department of Fisheries and Wildlife, Oregon State University, Corvallis, Oregon, United States of America; 2 Eastern Oregon Agriculture and Natural Resource Program, Oregon State University, Union, Oregon, United States of America; 3 USDA Forest Service, Rocky Mountain Research Station, Missoula, Montana, United States of America; 4 Wyoming Game and Fish Department, Lander, Wyoming, United States of America; 5 U.S. Geological Survey, Oregon Cooperative Fish and Wildlife Research Unit, Department of Fisheries and Wildlife, Oregon State University, Corvallis, Oregon, United States of America; Institute of Zoology, CHINA

## Abstract

Grassland and shrubland birds are declining globally due in part to anthropogenic habitat modification. Because population performance of these species is also influenced by non-anthropogenic factors, it is important to incorporate all relevant ecological drivers into demographic models. We used design-based sampling and occupancy models to test relationships of environmental factors that influence raptor demographics with re-occupancy of breeding territories by ferruginous hawks (*Buteo regalis*) across Wyoming, USA, 2011–2013. We also tested correlations of territory re-occupancy with oil and gas infrastructure—a leading cause of habitat modification throughout the range of this species of conservation concern. Probability of re-occupancy was not related to any covariates we investigated in 2011, had a strong negative relationship with cover of sagebrush (*Artemisia* spp.) in 2012, was slightly higher for territories with artificial platforms than other nest substrates in 2013, and had a positive relationship with abundance of ground squirrels (*Urocitellus* spp.) that was strong in 2012 and weak in 2013. Associations with roads were weak and varied by year, road-type, and scale: in 2012, re-occupancy probability had a weak positive correlation with density of roads not associated with oil and gas fields at the territory-scale; however, in 2013 re-occupancy had a very weak negative correlation with density of oil and gas field roads near nest sites (≤500 m). Although our results indicate re-occupancy of breeding territories by ferruginous hawks was compatible with densities of anthropogenic infrastructure in our study area, the lack of relationships between oil and gas well density and territory re-occupancy may have occurred because pre-treatment data were unavailable. We used probabilistic sampling at a broad spatial extent, methods to account for imperfect detection, and conducted extensive prey sampling; nonetheless, future research using before-after-control-impact designs is needed to fully assess impacts of oil and gas development on ferruginous hawks.

## Introduction

Habitat heterogeneity can regulate animal populations through effects on individual fitness, survival, and reproduction [1]. Quality of breeding habitat may be especially important to population performance because of its influence on reproductive output [2]. Numerous factors contribute to the quality of breeding habitat, including food availability, vegetation characteristics, predation risk, weather, and climate, all of which may be affected by anthropogenic activities [3]. It is, therefore, important to account for the influence of both ecological and anthropogenic factors when examining the effects of breeding habitat quality on wildlife demographics [4, 5].

Breeding avifauna of North American grassland and sagebrush steppe ecosystems experienced declines during the 20^th^ century [6, 7] associated with widespread anthropogenic modification of these habitats [8]. Agricultural tillage of native grasslands is the primary factor implicated in historical declines [6]. More recently, however, oil and gas development in the western U.S. has expanded disproportionately in grassland and shrubland ecosystems [9], where it has negatively impacted some breeding birds [10, 11].

The ferruginous hawk (*Buteo regalis*) is a raptor species of conservation concern that inhabits the arid grassland and shrubland regions of western North America [12]. Documented range contractions [13] and a reputation for sensitivity to anthropogenic disturbance [14, 15] have led to various conservation designations for the ferruginous hawk across its range [12], including Threatened status under the Species at Risk Act in Canada [16], and an unsuccessful petition for listing as Threatened under the Endangered Species Act in the U.S. [17].

Suitable breeding habitat for ferruginous hawks has been characterized by abundant mammalian prey [18], sparse vegetation [19], low congeneric competition [20], and availability of elevated nesting substrates [21] that may reduce predation risk [22, 23]. Ferruginous hawks may benefit from some anthropogenic sources of landscape heterogeneity, like roads not associated with oil and gas development [22, 24], increased abundance of mammalian prey along habitat edges [23, 24], and anthropogenic structures used for perching and nesting [23], including artificial nest platforms installed for mitigation or habitat enhancement [25]. Ferruginous hawks have, however, been negatively impacted by fragmentation of native habitat [26] from agricultural tillage [27] and oil and gas development [28, 29]. In addition to loss of habitat, fragmentation may facilitate disturbance of ferruginous hawks at nesting sites [14, 15], including illegal shooting [30]. Although relatively little agricultural tillage has occurred in Wyoming [31], where our study took place, the number of oil and gas wells in the state has more-than doubled since 2000 [32]. The rapid expansion of oil and gas development in Wyoming has occurred almost exclusively in grassland and shrubland habitats [33], creating a pressing need to better understand the relative importance of natural and anthropogenic factors in determining habitat quality for shrubland and grassland obligates such as the ferruginous hawk.

Our objectives were to determine which environmental factors most influenced quality of breeding habitat for ferruginous hawks, and to identify potential impacts from oil and gas development in the context of other factors known to influence population performance of raptors. Specifically, we tested whether re-occupancy of breeding territories was associated with prey abundance, vegetative cover, density of oil and gas infrastructure, and characteristics of nest substrates. An additional goal was to conduct our study at a broad spatial extent relevant to management (i.e. the distribution of ferruginous hawks in Wyoming) using probabilistic sampling and occupancy models because previous studies of ferruginous hawks have been conducted at finer scales and/or used samples of convenience, which are known to produce biased results [34, 35].

## Methods

### Study area

**Fig 1:**
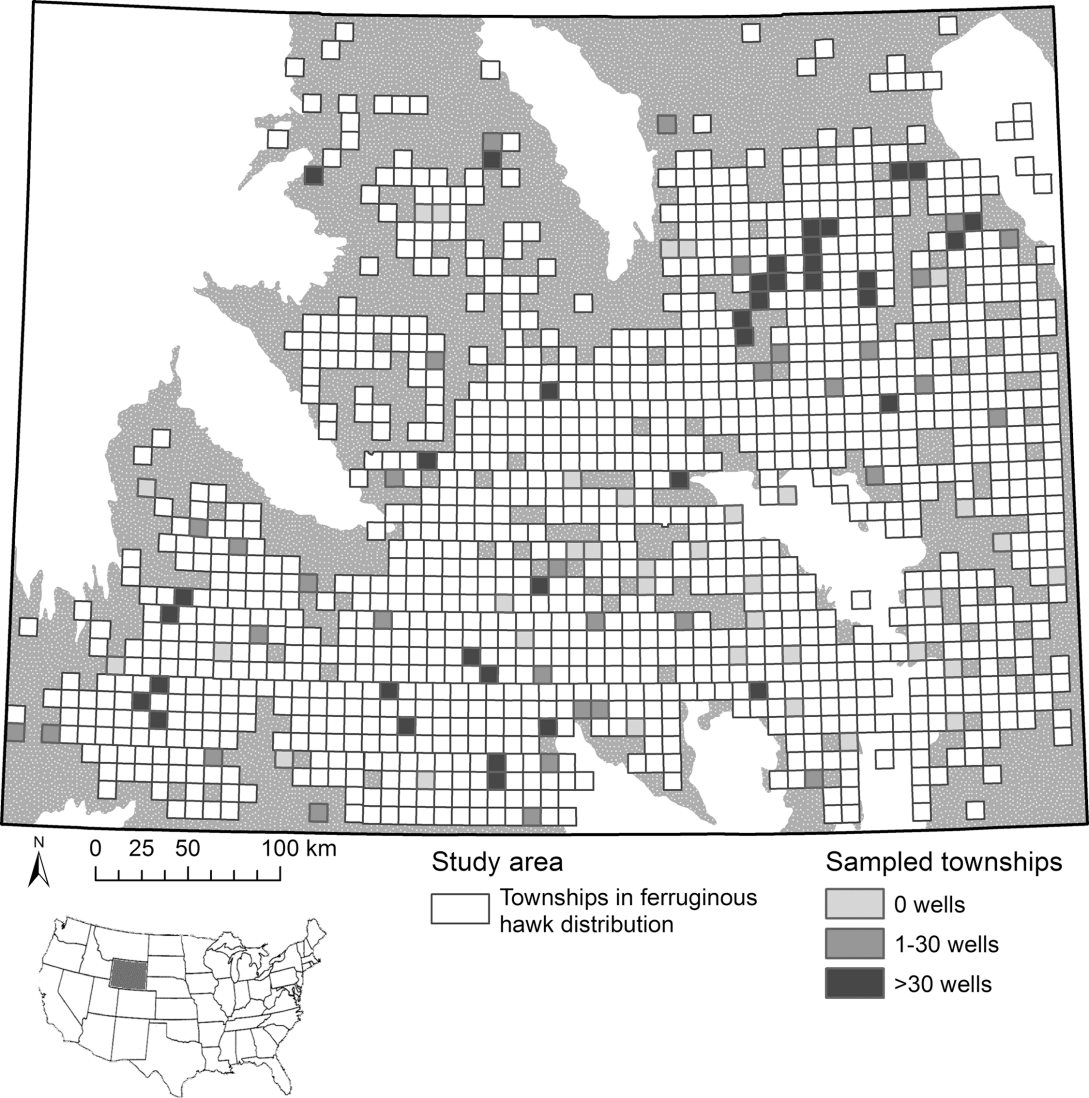
Study area encompassing the distribution of ferruginous hawks in Wyoming, USA. Depicted are Public Land Survey System townships with centroids in the study area and townships sampled for ferruginous hawk nests stratified by 3 levels of oil and gas well density. Grey areas are lowland basins and white areas are mountain ranges. Inset shows the location of Wyoming in the United States.

We defined our study area as the 1230 townships in the state of Wyoming, USA, with centroids in the distribution of ferruginous hawks [36] or with records of breeding ferruginous hawks from state databases (Fig 1). A township is an approximately square, 93.3-km^2^ area delineated by the U.S. Public Land Survey System [37]. Our study area included 114 217 km^2^, or approximately 45% of Wyoming, and was considerably larger than previous studies of ferruginous hawks relative to oil and gas development (e.g., 44 425 km^2^ [22], 2365 km^2^ [23], 171 km^2^ [24]). This area was dominated by sagebrush steppe (53%) and prairie (23%), with lesser areas of desert shrub and grassland (10%), and other (10%) vegetation types, and minimal agricultural (2%) and urban development (2%) [38]. Elevation ranged from approximately 1000 m to 2000 m, and mean annual precipitation from 15 cm to 40 cm [31].

All field work was conducted on public lands administered by the Bureau of Land Management, U.S. Forest Service, and state of Wyoming that did not require permits for access, and on private lands for which we obtained permissions to access property from 75 individual landowners. Although ferruginous hawks are protected under the Migratory Bird Treaty Act (16 U.S.C. §§ 703–712), no permits were required to conduct our study (including Institutional Animal Care and Use Committee approval) because we did not physically capture or handle animals.

### Nest survey

During spring of 2010 and 2011, we used fixed-wing aircraft (Bellanca Scout and Piper PA 18) to survey raptor nests in 104 townships in our study area. To ensure our sample represented the range of oil and gas development intensity across Wyoming, we stratified our study area by 3 densities of active oil and natural gas wells per township (none: 0 wells; low: 1−30 wells; and high: ≥31 wells). We then randomly selected 33 townships from each of these 3 strata and added 5 supplementary townships that had been part of previous survey efforts. We surveyed each township with 16 parallel north-south transects, 9.65 km in length, spaced 600 m apart, travelling at groundspeeds of 130 km/h, 60 m above ground level. A pilot and observer visually scanned from both sides of the plane and used a global positioning system (GPS) to record locations of occupied and unoccupied raptor nests within township boundaries, as well as nests detected while traveling between survey areas. Surveys were conducted by 3 different crews, each consisting of 1 pilot and 1 observer. This method enabled us to rapidly obtain a random sample of confirmed ferruginous hawk nests from a broad and rugged area without issues common to ground-based surveys, including bias introduced by observing from roads and access to private land [39].

### Territory re-occupancy survey

From 2011–2013 we monitored re-occupancy of ferruginous hawk breeding territories identified during nest surveys. Occupancy probability is a measure of the likelihood that an object or state of interest occurs in a sample unit during a period of time [40]. For this study, we defined re-occupancy as the probability that a ferruginous hawk occurred in association with a nest structure on a previously occupied territory during the breeding period. We adopted the term “re-occupancy” to reflect the fact that we monitored only sites that were occupied by ferruginous hawks when located in 2010 or 2011, which limited our scope of inference to territories with a recent history of occupancy. Although we considered using a grid-based patch-occupancy design, low densities of ferruginous hawk breeding territories would likely have resulted in prohibitively small sample sizes [21]. Although occurrence may be a misleading measure of habitat quality for some organisms [41], we chose territory re-occupancy as our response variable because occupancy and re-occupancy rates of raptors have been shown to correlate with factors that influence population performance, including breeding success, annual productivity, safety of nesting sites, and mortality risk [42, 43]. Since detection probability of raptors [21, 44, 45] and other organisms [46] is commonly <1, we used models developed by Mackenzie et al. [46] to estimate detection probability jointly with re-occupancy. Failure to account for detection probability is expected to create a negative bias in estimates of occupancy probability [46] and may produce misleading conclusions on covariate associations [47].

Our sample units were putative breeding territories, defined as circular buffers (7.06-km^2^ area, 1.5-km radius) around the nest site that was occupied when we located each territory. We based the size of territories on the average home range reported for ferruginous hawks (7.0 km^2^) in a summary by Olendorff [13]. We retained for re-occupancy monitoring only sites that were occupied by ferruginous hawks when located during the nest survey because we could not assign unoccupied nests to a single raptor species with certainty. We defined the time period for re-occupancy as the breeding period of ferruginous hawks in Wyoming, from arrival of this partially migratory species in early April to fledging of young in mid-July [12]. We classified occupancy states of territories according to Steenhof and Newton [48], except we did not consider a single adult with a nest showing signs of recent repair (e.g., green vegetation or freshly broken sticks) as evidence of re-occupancy because we could not reliably discern age of nest repairs from aircraft. Our minimum criteria for re-occupancy were, therefore, an individual in incubating position or a territorial pair observed with a nest.

We began re-occupancy surveys after ferruginous hawks had arrived on breeding territories in mid-April, and conducted repeat visits during the early breeding period in April and May to minimize violation of the closure assumption of single-season occupancy models [46]. We used removal sampling, in which surveys at a site stopped for the season after re-occupancy was detected, or a maximum of 3 visits had been completed. Surveys were conducted from small fixed-wing aircraft (Cessna 206), with a pilot and observer visually scanning from both sides of the plane. Surveys began with scan of the territory from approximately 200 m above ground level, followed by visits to all known nest structures, and a series of lower (30–60 m above ground level) passes over all potential nesting substrates within the territory. Speed (approx. 100 km/h ground speed), and search time (5–10 minutes) varied between sites based on habitat complexity. All surveys during 2012 and 2013 were conducted by the same observer and pilot. During 2011, survey occasions 1 and 2 were conducted by 3 pilot-observer crews, with the same observer as 2012 and 2013 conducting >1/3 of surveys on the 1^st^ occasion, >1/2 of surveys on the 2^nd^ occasion, and all surveys on the 3^rd^ occasion.

### Covariate data collection, estimation, and predictions

We extracted covariates from raster and vector data layers using ArcGIS v.10 (hereafter GIS) [49]. We present summary statistics of covariates in S1 Appendix. We estimated all covariates over the extent of territories (7.06 km^2^), with the exception of nest covariates, which refer to the most recently used substrate, and sampling strata, which refer to the extent of townships (93.3 km^2^). We estimated covariates related to oil and gas development at an additional finer extent (500-m radius around central nest site) based on approximate flight initiation distances of ferruginous hawks exposed to experimental disturbance treatments [14, 15] and recommended buffers for protection of raptor nests [50]. Although some studies document shorter flight initiation distances (e.g. 110 m [27]), we did not have sufficient sample size of nests near infrastructure to test relationships at extents finer than 500 m. Covariates were standardized internally in Program MARK [51].

We conducted extensive distance sampling of mammalian prey species using point and line transects during 2010–2012. We used open-population models [52] to define covariates representing detection-adjusted estimates of abundance for ground squirrels (*Urocitellus* spp.), and leporids (cottontails, *Sylvilagus* spp.; and white-tailed jackrabbits, *Lepus townsendii*). This approach allowed us to create spatially explicit models of prey abundance across the distribution of ferruginous hawks in Wyoming, although estimates were not year-specific (details of prey sampling and models are included in S2 Appendix). We hypothesized re-occupancy probability would be positively related to abundance of ground squirrels and leporids because productivity of ferruginous hawks increases with prey availability [18, 53].

We used a spatial model by Homer et al. [54] to define a covariate representing estimated percent cover of sagebrush (*Artemisia* spp.), the dominant shrub in our study area, as an index of vegetative cover. We predicted that re-occupancy probability would be negatively related to sagebrush cover because ferruginous hawks are associated with sparse vegetation throughout their range [12].

We used data from the Wyoming Oil and Gas Conservation Commission [32] to define covariates representing density of active oil and gas well pads. We hypothesized re-occupancy probability would be negatively related to well pad density because wells, associated infrastructure, and activities would disturb nesting behavior and habitat [14, 28, 29]. We predicted negative correlations would be stronger at the 500-m extent because disturbance closer to nest sites would be more likely to affect reproductive success and re-occupancy [14]. We stratified our initial sample by density of active oil and natural gas wells to ensure it represented the range of development intensity across Wyoming and to increase sample size of nest sites near active oil and gas wells. Although we did not predict re-occupancy would be negatively associated with well density at the relatively broad extent of townships, we included a categorical covariate representing the well density strata in our analysis to account for potential effects of this structure from our sampling design. We updated roads data from the Bureau of Land Management by heads-up digitizing and classifying additional roads using GIS and aerial imagery [55]. We used these data to define covariates representing length (km) of hard-packed, aggregate, or paved roads leading to active oil and gas well pads, including Bureau of Land Management and U.S. Forest Service roads improved for access to oil and gas fields. We also included covariates representing length (km) of hard-packed, aggregate, or paved roads not associated with oil and gas fields. We excluded unimproved “2-track” roads (including unmaintained roads to abandoned oil and gas well pads) from analyses because we were unable to reliably distinguish them using aerial imagery and did not predict they would influence re-occupancy. We hypothesized that re-occupancy probability would be negatively related to increasing length of both road types because roads would facilitate anthropogenic disturbance and fragment habitat [13, 56, 57]. We predicted negative correlations would be strongest at the 500-m extent from roads in closer proximity to nest sites.

We made ocular estimates of nest height and classified nest substrates annually during aerial surveys, and ground visits when possible. We used these data to define a covariate representing estimated height above ground (m) of nest site. We predicted that re-occupancy probability would be positively related to nest height because ferruginous hawks are more likely to use taller nest sites [58, 59], possibly due to security from mammalian [22] and avian predators [29, 59]. Additionally, we defined 3 categories of nest substrates we hypothesized would be related to re-occupancy probability: ground nests, natural elevated structures (trees, cliffs, and rock outcrops), and artificial nest structures. We modeled these categories in combinations representing 2 hypotheses: first, we compared all 3 categories with the prediction that natural elevated structures and artificial nest structures would have unique re-occupancy probabilities greater than ground nests [58, 59, 60]; second, we constrained natural elevated and ground nests to be equal, representing the hypothesis that artificial nest structures would have higher re-occupancy probability than all other substrate types [22, 61].

For detection probability, we defined a group effect representing 4 categories of substrates previously reported to influence detection of ferruginous hawk nests on aerial surveys in Wyoming [44], and modified to include additional substrates in our study area: (1) rock pile without shrubs, exposed soil hill, rock pedestal, cliff-spur tip; (2) cliff side, ground with sparse or no shrubs; (3) lone tree, artificial nest platform, other anthropogenic structure; and (4) tree groves, rock pile with shrubs. We hypothesized that detection probability would have the same relationship to nest substrates observed by Ayers and Anderson [44]. We also predicted that detection probability would be positively related to nest height because taller structures would be more visually prominent.

### Model set and selection

We estimated probabilities of detection (*p*) and re-occupancy (*ψ*) using single-species, single-season occupancy models [46] in Program MARK [51]. High re-occupancy rates and relatively few extinction and colonization events, combined with a short time series (3 years of survey data) made multi-season occupancy models unfeasible. We could not evaluate effects of observers or other factors that varied between survey occasions on *p* because estimation of occasion-specific detection probabilities is not technically possible under a removal sampling design [46]. Thus, we tested models for *p* as a function of annual covariates (nest substrate and height), an additive linear time trend across survey occasions (*T*), and constant (.).

To avoid running large numbers of models associated with all combinations of covariates, we used a sequential approach (e.g., [62–65]) to create separate models for each year. We (1) selected the top model for *p* from additive combinations of individual covariates and a linear time trend while *ψ* was held constant (i.e. intercept-only); (2) used the top model structure for *p* to select competitive individual covariates for *ψ* within each covariate category; (3) combined competitive predictors from each category into a final set of models with additive combinations of 1–4 covariates for *ψ* and the top structure for *p* from stage 1; and (4) verified the structure for *p* was still appropriate by substituting competitive forms from stage 1 into top models from stage 3. We included the intercept-only structure for *p* during stage 1 and *ψ* at all stages. We tested for correlations between covariates, and did not include those that were highly correlated (*R* > |0.6|) together in the same additive models. We ranked models using the Akaike Information Criterion adjusted for small sample size (AIC_*c*_) [66]. At each stage we advanced models that were within 2 AIC_*c*_ of the top model. We interpreted models that were within 2 AIC_*c*_ of the top model and did not contain uninformative parameters, per Arnold [67], to have strong support, and evaluated group means, standardized covariate point estimates ($\hat{\beta }$), and their 95% confidence intervals (CI) as indicators of the direction and strength of relationships. We interpreted lack of overlap between 95% CI of group means, or coefficient CI and zero, as evidence of strong relationships. When CI of group means overlapped, or coefficient CI overlapped zero, we interpreted the degree of asymmetrical overlap to indicate the relative strength of relationships [68].

## Results

### Re-occupancy probabilities

We located 67 ferruginous hawk territories during nest surveys in 2010, which we monitored for re-occupancy during 2011. Nest surveys in 2011 resulted in the addition of 38 territories, for a total sample of 105 territories monitored for re-occupancy during 2012 and 2013.

**Fig 2:**
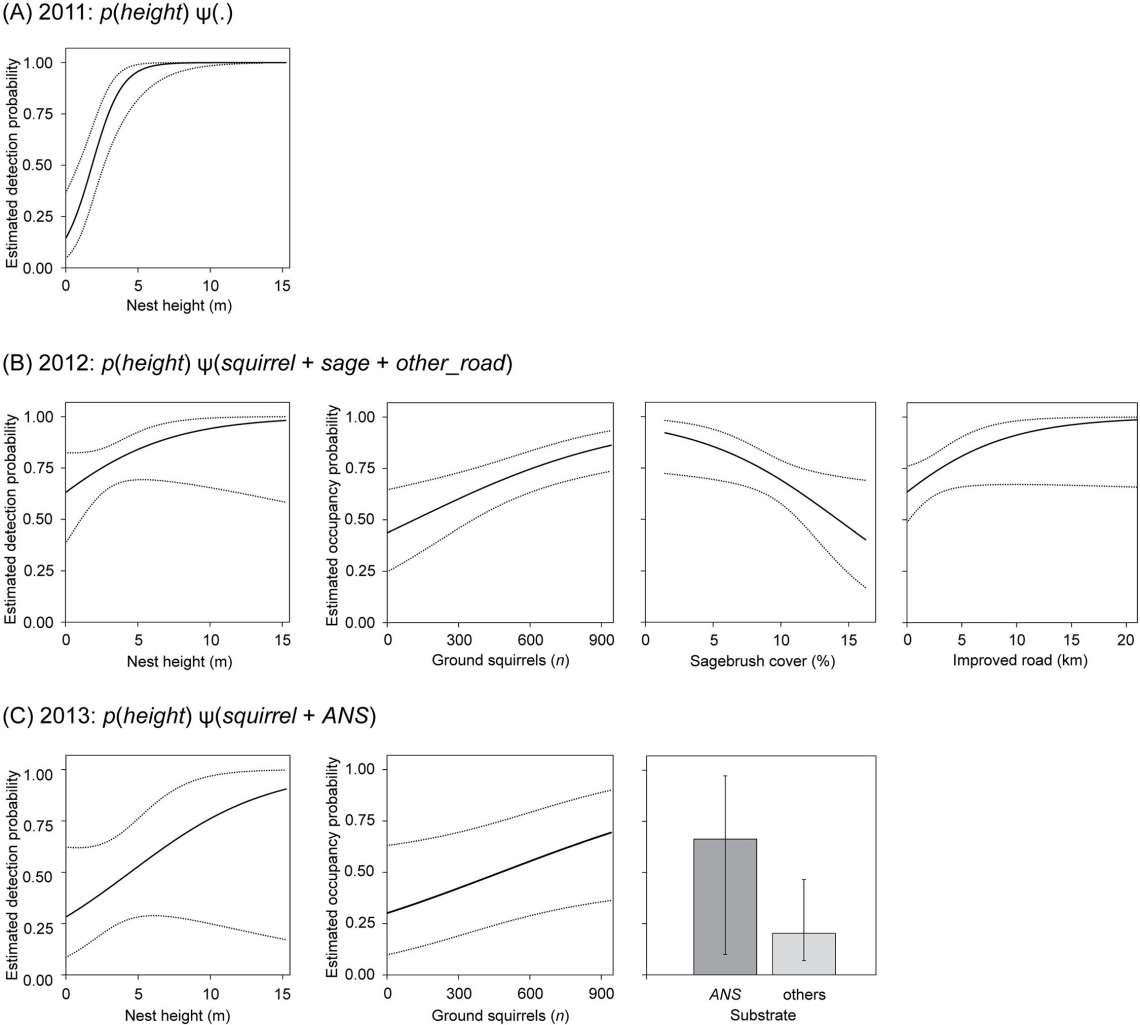
Relationships of covariates with detection and re-occupancy probabilities of ferruginous hawk territories in Wyoming, USA. Plots depict probabilities of detection (*p*) and re-occupancy (*ψ*) as functions of individual covariates from best-approximating single-season models with other covariates fixed at mean values during (A) 2011, (B) 2012, and (C) 2013. Numerical covariate relationships are illustrated as functions (black lines) with 95% CI (dotted lines), and categorical covariates as group means (bars) with 95% CI (error bars). Model selection results are presented in Table 1. Covariates are defined as follows. Subscripts indicate spatial extent of 500-m radius around central nest site; covariates without subscripts refer to the extent of putative territory (1.5-km radius), except *height*, which refers to the most recently used substrate. Covariates are defined as follows: *height*, height (m) of nest substrate; *squirrel*, abundance of ground squirrels (*Urocitellus* spp.); *sage*, cover (%) of sagebrush (*Artemisia* spp.); *gas_road*_*500*_, length (km) of roads associated with oil and gas fields; *other_road*, length (km) of other improved roads; *ANS*, categorical covariate representing artificial nest structures compared to all other substrates.

Re-occupancy by ferruginous hawks was not related to any covariates we investigated in 2011. In 2012, re-occupancy probability had a strong positive relationship to ground squirrel abundance ($\hat{\beta }$_*squirrel*_ = 0.71, 95% CI = 0.27–1.16), a strong negative relationship to percent cover of sagebrush ($\hat{\beta }$_*sage*_ = –0.58, 95% CI = –1.09 to –0.07), and a weak positive relationship to length of improved roads not associated with oil and gas developments at the territory-extent ($\hat{\beta }$_*other_road*_ = 0.65, 95% CI = –0.03 to 1.34; Fig 2). In 2013, re-occupancy probability had a weak positive relationship to ground squirrel abundance ($\hat{\beta }$_*squirrel*_ = 0.56, 95% CI = –0.01 to 1.13), and was higher for artificial nest structures ($\hat{\psi }$ = 0.66, 95% CI = 0.10–0.97) compared to other nest substrates ($\hat{\psi }$ = 0.20, 95% CI = 0.07–0.47), although strong overlap of coefficient 95% CI with zero ($\hat{\beta }$_*ANS*_ = 0.90, 95% CI = –0.27 to 2.07) suggest this relationship was not important (Fig 2).

We found no significant differences in re-occupancy probability by township-level well-density strata. A competitive model in 2013 (*p*_*height*_ *ψ*_*gas_road500 + ANS*_; Table 1) included a negative relationship of re-occupancy probability to length of oil and gas field roads within 500 m of nest sites ($\hat{\beta }$_*gas_road500*_ = –0.60, 95% CI = –1.42 to 0.22), but strong overlap of coefficient 95% CI with zero suggest this relationship was not important. We did not consider covariate relationships in other competitive models (Table 1) to be important to ferruginous hawk site re-occupancy because all were versions of best-approximating models with additional covariates that did not decrease deviance enough to improve AIC_*c*_ over reduced parameter models, and had coefficient 95% CI that included zero, suggesting weak associations [67]. Probability of territory re-occupancy from top models did not differ significantly during 3 years of monitoring (2011: $\hat{\psi }$ = 0.72, 95% CI = 0.57–0.83; 2012: $\hat{\psi }$ = 0.74, 95% CI = 0.62–0.82; 2013: $\hat{\psi }$ = 0.54, 95% CI = 0.28–0.78).

**Table 1:** Competitive models of detection and re-occupancy probabilities for ferruginous hawks in Wyoming, USA.

Year	Model Structure	AIC_*c*_	ΔAIC_*c*_	*w*_*i*_	*K*	Deviance
**2011**	***p*(*height*) *ψ*(.)**	134.02	0.00	0.16	3	127.63
	*p*(*height*) *ψ*(*strata*)	134.07	0.06	0.15	5	123.09
	*p*(*height*) *ψ*(*ANS*)	135.43	1.41	0.08	4	126.78
	*p*(*height*) *ψ*(*squirrel + ANS + strata*)	135.80	1.79	0.06	7	119.90
	*p*(*height*) *ψ*(*sage + ANS + strata*)	135.84	1.82	0.06	7	119.94
	*p*(*height*) *ψ*(*leporid + ANS + strata*)	135.93	1.91	0.06	7	120.03
	*p*(*height*) *ψ*(*squirrel*)	135.94	1.92	0.06	4	127.29
	*p*(*height*) *ψ*(*sage*)	135.95	1.94	0.06	4	127.31
	*p*(*height*) *ψ*(*leporid*)	135.99	1.98	0.06	4	127.35
**2012**	***p*(*height*) *ψ*(*squirrel* + *sage* + *other_road*)**	214.98	0.00	0.33	6	202.12
	*p*(*height*) *ψ*(*squirrel* + *sage* + *other_road* + *ANS*)	216.00	1.02	0.20	7	200.85
**2013**	***p*(*height*) *ψ*(*squirrel* + *ANS*)**	217.29	0.00	0.14	5	206.69
	*p*(*height*) *ψ*(*squirrel* + *ANS* + *gas_road*_*500*_)	217.77	0.48	0.11	6	204.91
	***p*(*height*) *ψ*(*gas_road***_***500***_ **+ *ANS*)**	218.24	0.95	0.09	5	207.64
	*p*(*height*) *ψ*(*squirrel* + *ANS* + *wellpads*_*500*_)	218.50	1.21	0.07	6	205.65
	*p*(*height*) *ψ*(*squirrel* + *ANS* + *other_road*)	218.50	1.21	0.07	6	205.65

Model parameters are probabilities of detection (*p*) and re-occupancy (*ψ*). Provided for each model are values of the Akaike Information Criterion adjusted for small sample sizes (AIC_*c*_), ΔAIC_*c*_, Akaike weight (*w*_*i*_), number of parameters (*K*), and deviance. **Bold text** denotes models without uninformative parameters, per Arnold [67]. Definitions of covariates are provided as follows. Subscripts indicate spatial extent of 500-m radius around central nest site; covariates without subscripts refer to the extent of putative territory (1.5-km radius), except nest covariates refer to the most recently used substrate, and *strata* refers to the extent of townships (93.3 km^2^). Covariates are defined as follows: *squirrel*, abundance of ground squirrels (*Urocitellus* spp.); *leporid*, abundance of leporids (*Sylvilagus* spp. and *Lepus townsendii*); *sage*, cover (%) of sagebrush (*Artemisia* spp.); *strata*, categorical covariate representing density of active oil and natural gas wells per township from stratification of initial nest survey (none: 0 wells; low: 1−30 wells; and high: ≥31 wells); *wellpads*_500_, number of active oil and gas well pads; *gas_road*_*500*_, length (km) of roads associated with oil and gas fields; *other_road*, length (km) of other improved roads; *height*, estimated height (m) of nest substrate; *ANS*, categorical covariate representing artificial nest structures compared to all other substrates.

### Detection probabilities

Nest height was the top predictor of detection probability in all years; this relationship was strong in 2011 ($\hat{\beta }$_*height*_ = 2.76, 95% CI = 1.42–4.09), and weak in 2012 ($\hat{\beta }$_*height*_ = 0.60, 95% CI = –0.17 to 1.38) and 2013 ($\hat{\beta }$_*height*_ = 0.56, 95% CI = –0.28 to 1.39). We did not test this explicitly, but the relationship of detection to nest height in 2011 appeared non-linear: detection probability increased from 0.14 to 0.98 as nest height increased from 0 m to 6 m, but detection stabilized at >0.99 for nests with heights from 6 m to 15 m (Fig 1). Per visit detection probabilities varied between years, with detection probability in 2011 ($\hat{p}$ = 0.79, 95% CI = 0.60–0.91) and 2012 ($\hat{p}$ = 0.78, 95% CI = 0.67–0.87) higher than 2013 ($\hat{p}$ = 0.44, 95% CI = 0.23–0.66), from top models with mean covariate values. The probability that ferruginous hawks were detected during at least 1 of 3 visits ($\hat{p}*$; $1-{\left(1-\hat{p}\right)}^{3}$) were high: ≥0.99 in 2011 and 2012, and 0.82 in 2013.

## Discussion

### Relationships with re-occupancy probabilities

Our results supported several of our hypotheses on relationships of territory re-occupancy to prey abundance, nest substrates, and vegetation. Contrary to our predictions, however, re-occupancy was not strongly related to density of oil and gas infrastructure: ferruginous hawks used breeding territories that contained oil and gas roads and well pads, and density of infrastructure in territories did not affect the probability that they were re-occupied in subsequent years. While our results are consistent with previous research suggesting ferruginous hawks and other raptors were unaffected [24] or habituated to oil and gas development [57], we did not observe the positive association of ferruginous hawks with oil and gas fields reported elsewhere [23, 69]. We found only very weak evidence to suggest re-occupancy rates were lower for ferruginous hawk territories with more oil and gas field roads ≤500 m from nest sites. Contrary to our predictions, probability of re-occupancy also had a weak positive correlation with improved roads not associated with oil and gas fields at the territory-extent. Though weakly supported in our study, these results are consistent with previous research suggesting direction of effects of roads on raptors changed with road type and scale [57]. Nest sites close to roads may be occupied less frequently due to greater disturbance [13, 57], while at a broader scale, roads can modify habitat to the benefit of some raptors [70–72] and their prey [24].

Ground squirrels of the genus *Urocitellus* are important in the diets of ferruginous hawks [73–75], and our results suggest ground squirrel abundance influenced re-occupancy of territories. Lack of correlation between leporid abundance and re-occupancy for ferruginous hawks may have been related to a cyclic low in leporid populations in Wyoming during our study [76] or their contribution to the diet was limited. Although we conducted extensive surveys during the nesting season, prey abundance in early spring before our surveys may have affected re-occupancy more strongly, and our ability to detect relationships between re-occupancy and prey may also have been limited because our estimates of prey abundance were not specific to years or time periods within years [77].

Ferruginous hawks are associated with low vegetative cover throughout their range [12] and our results suggest relative scarcity of vegetation (as indexed by % sagebrush cover) can influence territory re-occupancy. Lack of vegetative cover increases accessibility of prey to foraging ferruginous hawks [19], and may also have been correlated with abundance of prey species not included in our study, specifically prairie dogs (*Cynomys* spp.) and pocket gophers (*Thomomys* spp.). Although management to reduce sagebrush cover has occurred in our study area, we are not aware of any affected ferruginous hawk territories in our sample. We therefore interpret this result to reflect higher probability of re-occupancy for ferruginous hawks in areas in which sagebrush naturally occurs at lower density, including badlands, playas, salt desert shrublands, and prairies.

We found some evidence to support previous research suggesting re-occupancy rates of ferruginous hawks were higher on artificial nest platforms than other substrates [22, 61]. Availability of substrates can limit densities of breeding raptors in non-forested habitats [20, 78], and most nest platforms in our study area were in territories with few other elevated structures.

### Relationships with detection probabilities

Our results affirm the importance of accounting for influences of year and site characteristics on detection of raptors. Though generally high, detection rates were <1, influenced by nest height, and varied between years. Height of ferruginous hawk nests was strongly correlated with detection probability during the first year of re-occupancy surveys when observers were naïve to nest site locations, and less strongly correlated in subsequent years. Lack of correlation between detection and substrate groups of Ayers and Anderson [44] may also have resulted from observers’ prior knowledge of nest site locations. We could not test correlations of weather with territory re-occupancy and detection among years because we modeled years separately; however, we speculate that the lower re-occupancy and detection rates for ferruginous hawks in 2013 were related to unusually high snow cover that spring.

### Future research

The lack of relationships between re-occupancy probability and covariates during the first year of our study (2011) was likely the result of smaller sample sizes and less time for territories to transition from their initial occupied state. Longer-term studies using multi-season occupancy models, larger sample sizes, and year-specific covariates could, thus, help clarify the relative importance of the different environmental factors that we found were related to re-occupancy between years. Future research could also benefit from longer data sets because disturbance may take years or generations to affect patterns of occupancy for long-lived organisms, like raptors, that exhibit high degrees of site-fidelity and natal philopatry [79]. In the short term, multi-state models could be used to integrate detection-adjusted estimates of reproductive success with occupancy models. We used density of anthropogenic infrastructure as an index of disturbance because of its direct effect on habitat; however, frequency and type of human activity (e.g. vehicle and foot traffic, noise levels) and direct measures of habitat loss [80] may have been more representative metrics. Additionally, studies could evaluate relationships with development at other spatial extents, including landscape-scale effects from outside territories, and finer-scale effects on movement and nest site selection within home ranges.

Despite stratification by density of oil and gas wells, our sample contained few territories (*n* = 12) with occupied nest sites ≤500 m from active well pads—the extent at which we predicted disturbance would be greatest. Because our study was conducted post-construction and monitored recently occupied territories, it is possible that negative impacts that might have been incurred when pairs initially selected territories were not captured. In addition, it is possible that occupied sites in close proximity to infrastructure had combinations of characteristics that maximized benefits and minimized costs for raptors occupying developed areas, or were occupied by individuals tolerant of disturbance. We therefore suggest before-after control-impact [81] and/or patch-based occupancy studies [82] are needed to make strong inference about the relationship of ferruginous hawks to oil and gas development. Our results will be a useful baseline for comparison as density of energy development continues to increase in our study area.

### Conservation implications

Our results suggest efforts to conserve ferruginous hawk nest sites and habitat should be focused on areas with abundant ground squirrels and low natural sagebrush cover, and provide additional support for continued use of artificial nest platforms for habitat enhancement and to mitigate loss of natural nest substrates [83]. Our study occurred post-construction, and therefore could not address whether historical density or distribution of ferruginous hawks has been affected by oil and gas development. We were, however, able to evaluate the importance of oil and gas infrastructure relative to other environmental factors in determining patterns of re-occupancy for territories with a history of post-construction use. Our results suggest that re-occupancy of territories by ferruginous hawks was not negatively impacted by the density of oil and gas infrastructure observed during this study (well pads: mean = 0.67/km^2^, range = 0/km^2^ to 3.16/km^2^; oil and gas field roads: mean = 0.47 km/km^2^, range = 0 km/km^2^ to 3.54 km/km^2^). While our results indicate re-occupancy of territories by ferruginous hawks was compatible with densities of oil and gas infrastructure observed during this study, we emphasize that our findings do not imply territory re-occupancy is unaffected by higher densities of infrastructure, or that other aspects of ferruginous hawk behavior and ecology are not affected by oil and gas development.

We emphasize the importance and feasibility of using probabilistic sampling and methods that account for imperfect detection to monitor species of conservation concern at broad spatial extents. We suggest our monitoring approach and sample of territories will increase in value as longer time-series permit use of more complex, multi-season and multi-state models that can be useful for monitoring ferruginous hawks relative to multiple factors, including accelerating development of energy resources.

## Supporting Information

S1 Appendix Summary of covariate values for models of ferruginous hawk territory re-occupancy. (DOCX)

S2 Appendix Methods for estimation of mammalian prey abundance. (DOCX)
