# Supplementary material for: Re-Occupancy of Breeding Territories by Ferruginous Hawks in Wyoming: Relationships to Environmental and Anthropogenic Factors
Source: PLoS One. 2016 Apr 6;11(4):e0152977. doi: 10.1371/journal.pone.0152977 (PMC4822948; doi:10.1371/journal.pone.0152977)
Supplement: S1 Appendix — (DOCX) [file pone.0152977.s001.docx]

**Appendix S1. Summary of covariate values for models of ferruginous hawk territory re-occupancy.**

**Table S1A. Summary of numerical covariates used in models of territory re-occupancy by ferruginous hawks in Wyoming, USA, 2011–2013.** Provided are units, ranges, means, and standard errors (SE). Sample sizes were *n* = 67 in 2011, and *n* = 105 in 2012 and 2013. Covariates are defined below^a^. Categorical covariates are summarized in Table S1B. Covariates *wellpads* and *height* were annually-specific and others were estimated for the study period. Hypotheses are summarized in text.

| **Covariate** | **Units** | **Year** | | | | | | | | |
| --- | --- | --- | --- | --- | --- | --- | --- | --- | --- | --- |
|  |  | **2011** | | | **2012** | | | **2013** | | |
|  |  | **Range** | **Mean** | **SE** | **Range** | **Mean** | **SE** | **Range** | **Mean** | **SE** |
| *squirrel* | squirrels | 0.00–940.71 | 562.06 | 38.50 | 0.00–940.71 | 578.23 | 31.14 | 0.00–940.71 | 578.23 | 31.14 |
| *leporid* | leporids | 64.42–99.43 | 94.07 | 0.79 | 64.42–104.10 | 94.41 | 0.56 | 64.42–104.10 | 94.41 | 0.56 |
| *sage* | % | 1.45–16.25 | 8.85 | 3.22 | 1.50–16.00 | 8.90 | 0.30 | 1.50–16.00 | 8.90 | 0.30 |
| *wellpads* | wellpads | 0–22 | 2.99 | 0.74 | 0–22 | 5.57 | 0.54 | 0–23 | 5.60 | 0.55 |
| *wellpads_500_* | wellpads | 0–3 | 0.27 | 0.09 | 0–3 | 0.21 | 0.06 | 0–3 | 0.21 | 0.06 |
| *gas_road* | km | 0.00–25.02 | 3.68 | 0.78 | 0.00–25.02 | 3.11 | 0.57 | 0.00–25.02 | 3.11 | 0.57 |
| *gas_road_500_* | km | 0.00–8.33 | 0.69 | 0.19 | 0.00–11.10 | 0.64 | 0.16 | 0.00–11.10 | 0.64 | 0.16 |
| *other_road* | km | 0.00–13.84 | 2.65 | 0.42 | 0.00–21.31 | 2.68 | 0.36 | 0.00–21.31 | 2.68 | 0.36 |
| *other_road_500_* | km | 0.00–13.6 | 1.05 | 0.27 | 0.00–17.49 | 1.20 | 0.26 | 0.00–17.49 | 1.20 | 0.26 |
| *height* | m | 0.00–15.24 | 3.19 | 0.35 | 0.00–15.24 | 3.32 | 0.26 | 0.00–15.24 | 3.21 | 0.26 |

^a^ Subscripts indicate spatial extent of 500-m radius around central nest site; covariates without subscripts refer to the extent of putative territory (1.5-km radius), except *height*, which refers to the most recently used substrate. Covariates are defined as follows: *squirrel*, abundance of ground squirrels (*Urocitellus* spp.); *leporid*, abundance of leporids (*Sylvilagus* spp. and *Lepus* *townsendii*); *sage*, cover (%) of sagebrush (*Artemisia* spp.); *wellpads*, *wellpads*_500_, number of active oil and gas well pads; *gas_road*, *gas_road_500_*, length (km) of roads associated with oil and gas fields; *other_road*, *other_road_500_*, length (km) of other improved roads; *height*, height (m) of nest substrate.

**Table S1B. Number of ferruginous hawk breeding territories in categories of nest substrates predicted to influence detection and re-occupancy probabilities in Wyoming, USA, 2011–2013.** We defined 3 covariate groups to predict re-occupancy probability: *ground*, ground nests; *natural*, natural elevated structures; *ANS*, artificial nest structures; and 4 groups for detection probability based on Ayers and Anderson (1999): *nest1*, rock pile without shrubs, exposed soil hill, rock pedestal, cliff-spur tip; *nest2*, cliff side, ground with sparse or no shrubs; *nest3*, lone tree, artificial nest platform, other anthropogenic structure; and *nest4*, tree groves, rock pile with shrubs. These counts reflect the most recently used substrate in a territory. Hypotheses are summarized in text.

| **Covariate Levels** | **Year** | | |
| --- | --- | --- | --- |
|  | **2011** | **2012** | **2013** |
| *ground* | 16 | 22 | 23 |
| *natural* | 32 | 55 | 55 |
| *ANS* | 19 | 28 | 27 |
| *nest1* | 29 | 29 | 29 |
| *nest2* | 10 | 10 | 10 |
| *nest3* | 56 | 56 | 56 |
| *nest4* | 10 | 10 | 10 |

**Table S1C. Number of ferruginous hawk breeding territories in townships stratified by number of active oil and natural gas wells in Wyoming, USA, 2011–2013.** To ensure our sample represented the range of oil and gas development intensity across Wyoming, we stratified our study area by 3 densities of active oil and natural gas wells per township (none: 0 wells; low: 1−30 wells; and high: ≥31 wells). Stratified nest surveys were conducted during 2010 and 2011, and we monitored re-occupancy of the resulting sample of breeding territories during 2011–2013. A township is an approximately square, 93.3 km^2^ area delineated by the U.S. Public Land Survey System.

| **Well density stratum** | **Year** | | |
| --- | --- | --- | --- |
|  | **2011** | **2012** | **2013** |
| None (0 wells) | 30 | 46 | 46 |
| Low (1–30 wells) | 16 | 29 | 29 |
| High (≥31 wells) | 21 | 30 | 30 |
