# Supplementary material for: Re-Occupancy of Breeding Territories by Ferruginous Hawks in Wyoming: Relationships to Environmental and Anthropogenic Factors
Source: PLoS One. 2016 Apr 6;11(4):e0152977. doi: 10.1371/journal.pone.0152977 (PMC4822948; doi:10.1371/journal.pone.0152977)
Supplement: S2 Appendix — (DOCX) [file pone.0152977.s002.docx]

**Appendix S2. Estimation of mammalian prey abundance.**

**Methods**

During 2010–2012, we conducted distance sampling of mammalian prey species within ferruginous hawk territories and random locations within the study area described in the Methods section of the manuscript. Survey areas consisted of an annulus with an outer radius of 2 km and an inner radius of 0.5 km, centered on either a ferruginous hawk nest (*n* = 66) or a random location (*n* = 20) chosen using ArcGIS v.10 (hereafter GIS) [1]. Outer diameters of annuli corresponded to the approximate size of ferruginous hawk breeding territories and inner diameters were 0.5-km buffers to prevent disturbance of nesting hawks. Within each annulus, we used GIS to randomly place 6 line transects, 1 km in length, with 4 survey points at 333.3-m intervals along each line. We used point transects to sample sciurids because they are active and visible above ground [2, 3], and line transects for leporids because they generally must be flushed from concealed resting sites to be detected [4]. Sampling was conducted between 0630 and 1030 hrs when sciurids were active and leporids were sedentary. Point transects used a 5-min sampling interval, beginning with a brief 360° scan with the naked eye, and followed by a gradual scan using binoculars. Observers recorded the species of each animal detected and measured its distance and azimuth from the sampling point using a laser range finder and magnetic compass. Observers then used a handheld GPS to walk slowly (approx. 2 km/hr) to the next point, following the intervening line transect, and stopping to record the species, distance, and angle of each leporid encountered.

We used the ‘unmarked’ package in program R v. 3.0.1 [5] to model abundance separately for leporids and sciurids. The ‘unmarked’ package allows estimation of density from repeated sampling of a population in which detection is imperfect and temporary emigration may occur [6]. To account for lack of demographic closure between surveys, we modeled each visit as a primary occasion contributing to a combined estimate of abundance for the entire study period (2010–2012) that was not annually specific. We truncated all observations from point transects to 167 m to avoid accidental double detection between adjacent points.

We used a multi-step process to select best-approximating models of abundance and detection probability. First, we used distance modeling with no covariates for a null estimate of the number of each group at each transect, and considered 3 key functions (half-normal, hazard rate, exponential) and 2 distributions (Poisson and negative binomial) for each group’s null model. We ranked null models using the Akaike Information Criterion (AIC) [7], considering the model with the lowest AIC score to be the best-fitting key function and distribution for each prey group. After constructing a null model for each group, we considered a suite of covariates for abundance with detection probability held constant. Covariates for abundance included measures of vegetation, soils, climate, topography, and anthropogenic infrastructure (Table S2A) at 4 spatial extents (100m, 250m, 500m, 1000m) using a moving window approach [8]. To reduce the number of potential covariates for abundance, we evaluated univariate models for each variable at each extent, and selected the most predictive scale for each variable that ranked above the null model. We tested variables for multicollinearity using Pearson’s *R* > |0.7| and retained those with the lowest AIC scores when variables were correlated. We used the top-performing covariates for abundance to create a final set of candidate models that included covariates representing environmental factors we hypothesized would influence detection probability (i.e. topographic position index and shrub cover). We estimated all covariates for detection at the 250-m extent, corresponding to average maximum detection distances during surveys. We ranked final candidate models with AIC; if multiple models were within 2 ΔAIC of the top model, we averaged models in the confidence set to generate best estimates of prey abundance and detection probability. We used GIS to create spatial predictions of abundance for each prey group over the entire study area based on covariate coefficients from best-approximating models and corresponding spatial data layers.

**Results**

We sampled each site once during 2010 and twice during 2011 and 2012. We surveyed 32 sites during all 3 years, 51 sites during 2 years, and 3 sites during only 1 year. Our survey effort was extensive, covering 285 km of transects in 2010, 770 km in 2011, and 741 km in 2012. We detected 400 total leporids, comprising 140 white-tailed jackrabbits (*Lepus townsendii*), 68 desert cottontails (*Sylvilagus audobonii*), and 192 unidentified or other species. Based on results of null models, we selected the hazard rate key function with a negative binomial distribution as the best fitting detection function. The best multivariate model included proportion of sagebrush cover, shrub height, proportion of bare ground, average spring temperature, winter precipitation, spring precipitation, and minimum January temperature. Complete model selection results are included in Wallace [9]. This model predicted an average of 18.4 (SE = 6.03) leporids per km^2^ at surveyed sites. For the 7.06-km^2^ putative ferruginous hawk territories in our sample, this model predicted an average abundance of 94.07 (SE = 0.79) leporids in 2011 and 94.41 (SE = 0.56) leporids in 2012 and 2013 (S1 Appendix Table S1A).

We detected 3557 sciurids, consisting of 2925 Wyoming ground squirrels (*Urocitellus elegans*), 124 Uinta ground squirrels (*Urocitellus armatus*), and 508 unidentified or other species. Null model results suggested the half-normal key function with a negative binomial distribution was the best fitting detection function. The best-approximating multivariate model included topographic position index, distance to nearest road, spring precipitation, proportion of sagebrush cover, and proportion of bare ground. Complete model selection results are included in Wallace [9]. This model predicted an average of 120.16 (SE = 15.14) ground squirrels per km^2^ at surveyed sites. For the 7.06-km^2^ putative ferruginous hawk territories in our sample, this model predicted an average abundance of 562.06 (SE = 38.50) ground squirrels in 2011 and 578.23 (SE = 31.14) ground squirrels in 2012 and 2013 (S1 Appendix Table S1A).

References

1. ESRI (2011) ArcGIS Desktop: Release 10. Redlands: Environmental Systems Research Institute.

2. Schmutz JK, Flockhart DTT, Houston CS, McLoughlin PD. Demography of ferruginous hawks breeding in western Canada. J Wildl Manage. 2008;72: 1352-1360.

3. Andelt WF, White GC, Schnurr PM, Navo KW. Occupancy of random plots by white-tailed and Gunnison’s prairie dogs. J Wildl Manage. 2009;73: 35-44.

4. Wywialowski AP, Stoddart LC. Estimation of jack rabbit density: methodology makes a difference. J Wildl Manage. 1988;52: 57-59.

5. R Core Team. R: a language and environment for statistical computing. Vienna: R Foundation for Statistical Computing. Available: http://www.R-project.org. Accessed 1 January 2012.

6. Chandler RB, Royle JA, King DI. Inference about density and temporary emigration in unmarked populations. Ecology. 2011;92: 1429-1435.

7. Burnham KP, Anderson DR. Model selection and multi-model inference: a practical information-theoretic approach. New York: Springer; 2002.

8. Potvin F, Lowell K, Fortin MJ, Belanger L. How to test habitat selection at the home range scale: a resampling random windows technique. Ecoscience. 2001;8: 399-406.

9. Wallace ZP. Effects of oil and natural gas development on territory occupancy of ferruginous hawks and golden eagles in Wyoming, USA. M.Sc. Thesis, Oregon State University. 2014.

10. Homer CG, Aldridge CL, Meyer DK, Schell SJ. Multi-scale remote sensing sagebrush characterization with regression trees over Wyoming, USA: laying a foundation for monitoring. Int J Appl Earth Obs Geoinf. 2012;14: 233-244.

11. Reeves MC, Zhao M, Running SW. Applying improved estimates of MODIS productivity to characterize grassland vegetation dynamics. Rangeland Ecol Manag. 2006;59: 1-10.

12. Soil Survey Staff, Natural Resources Conservation Service, United States Department of Agriculture. U.S. General Soil Map: STATSGO2. Available: http://sdmdataaccess.nrcs.usda.gov. Accessed 2 July 2012.

13. PRISM Climate Group, Oregon State University. PRISM climate data. Available: http://prism.oregonstate.edu. Accessed 2 July 2012.

14. Gesch D, Evans G, Mauck J, Hutchinson J, Carswell WJ. The National Map—Elevation. U.S. Geological Survey Fact Sheet 2009-3053; 2009.

15. Weiss AD. Topographic positions and landforms analysis. San Diego: Proceedings of ESRI International User Conference, July 9–13, 2001.

16. Wyoming Oil and Gas Conservation Commission. WOGCC homepage. Available: http://wogcc.state.wy.us/. Accessed 5 June 2013.

**Table S2A. Covariates for abundance of sciurids and leporids in sagebrush steppe and prairie regions of Wyoming, USA, 2010–2012.** Presented are variable category, description, and source.

| **Category** | **Description** | **Source** |
| --- | --- | --- |
| Vegetation | % cover of bare ground | USGS Wyoming sagebrush products [10] |
|  | % cover of sagebrush (*Artemisia* spp.) |  |
|  | % cover of shrubs |  |
|  | % cover of herbaceous vegetation |  |
|  | Average shrub height |  |
|  | Annual normalized difference vegetation index from 2010 | MODIS data [11] |
| Soils | Soil texture (6 categories: fine to coarse) | US General soil map [12] |
|  | Soil depth |  |
| Climate | Precipitation amount in December and January | PRISM climate data [13] |
|  | Precipitation amount in April and May |  |
|  | Average temperature in April and May |  |
|  | Minimum temperature in January |  |
|  | Maximum temperature in July |  |
| Topographic | Elevation | National elevation map [14] |
|  | Topographic position index | Topographic position analysis [15] |
|  | Index of surface roughness |  |
| Anthropogenic | Distance to nearest road | Wyoming BLM data |
|  | Distance to nearest oil or gas well | Wyoming Oil and Gas Conservation Commission data [16] |
